# Laparoscopic Drainage of a Traumatic Intramural Duodenal Hematoma in a Child: A Case Report

**DOI:** 10.7759/cureus.86950

**Published:** 2025-06-29

**Authors:** Toshihito Uehara, Shin Shinyama, Hideo Kidogawa, Kohji Okamoto

**Affiliations:** 1 Department of Surgery, Kitakyushu City Yahata Hospital, Fukuoka, JPN

**Keywords:** duodenal hematoma, intramural hematoma, laparoscopic drainage, pediatric surgery, trauma

## Abstract

Intramural duodenal hematoma (IDH) is a rare event of blunt abdominal trauma in children. A 10-year-old female with IgA nephropathy sustained trauma and presented with duodenal obstruction secondary to IDH. Initial conservative management failed, prompting surgical intervention with laparoscopy. Intraoperatively, a 7.0 × 4.5 cm IDH was detected, and blood clot was evacuated. Postoperative recovery was uneventful. This case demonstrates that laparoscopic drainage can be a viable alternative for pediatric patients.

## Introduction

Intramural duodenal hematoma (IDH) in pediatric patients can be caused by various factors, with approximately 5% reported to result from blunt abdominal trauma. Due to the pliability of bones in children, blunt trauma is more likely to impact internal organs, and injuries to the liver, pancreas, and colon are relatively common. In contrast, duodenal injury is rare, occurring in approximately 0.01-0.7% of patients with blunt abdominal trauma, and in some cases, it may lead to the development of IDH [1-3]. The duodenum is anatomically fixed to the posterior abdominal wall and the ligament of Treitz. When subjected to blunt force, direct compression of the duodenum may lead to separation of the mucosal and submucosal layers, resulting in hematoma formation [1,4]. While many cases can be managed conservatively, surgical intervention is required when conservative treatment fails [5-8]. However, in pediatric cases, it is important to minimize surgical invasiveness. We report a case of IDH successfully treated with laparoscopic surgery.

## Case presentation

A 10-year-old female with a medical history of IgA nephropathy sustained blunt abdominal trauma while playing on a horizontal bar. At first, the abdominal pain was under control, but it gradually worsened, prompting her to present to our hospital one week post-injury. Physical examination revealed a palpable fullness, and mild tenderness was noted in the right upper quadrant. Laboratory investigations revealed no anemia (hemoglobin 15.1 g/dL). However, serum creatine kinase (CK) was elevated at 827 U/L, and blood urea nitrogen (BUN) was markedly elevated at 82.5 mg/dL (Table 1). The elevated BUN was suggestive of gastrointestinal bleeding due to trauma, and the elevated CK was indicative of muscle injury associated with the trauma.

**Table 1:** Laboratory data Serum CK and BUN levels were elevated. ALT, alanine aminotransferase; AST, aspartate aminotransferase; BUN, blood urea nitrogen; CK, creatine kinase; RBC, red blood cell, WBC, white blood cell

Test	Result	Reference range
WBC count (x10^3^/μL)	15.6	3.3-8.6
RBC count (x10^6^/μL)	5.41	3.86-4.92
Hemoglobin (g/dL)	15.1	11.6-14.8
Hematocrit (%)	43.7	35.1-44.4
Platelet count (x10^3^/μL)	489	158-348
Total protein (g/dL)	9.2	6.6-8.1
Total bilirubin (mg/dL)	1.1	0.4-1.5
AST (U/L)	46	13-30
ALT (U/L)	32	7-23
Lactate dehydrogenase (U/L)	327	124-222
Alkaline phosphatase (U/L)	242	38-113
CK (U/L)	827	41-153
BUN (mg/dL)	82.5	8.0-20.0
Creatinine (mg/dL)	1.15	0.46-0.79
Sodium (mmol/L)	137	138-145
Potassium (mmol/L)	4.4	3.6-4.8
Chloride (mmol/L)	88	101-108

**Figure 1:**
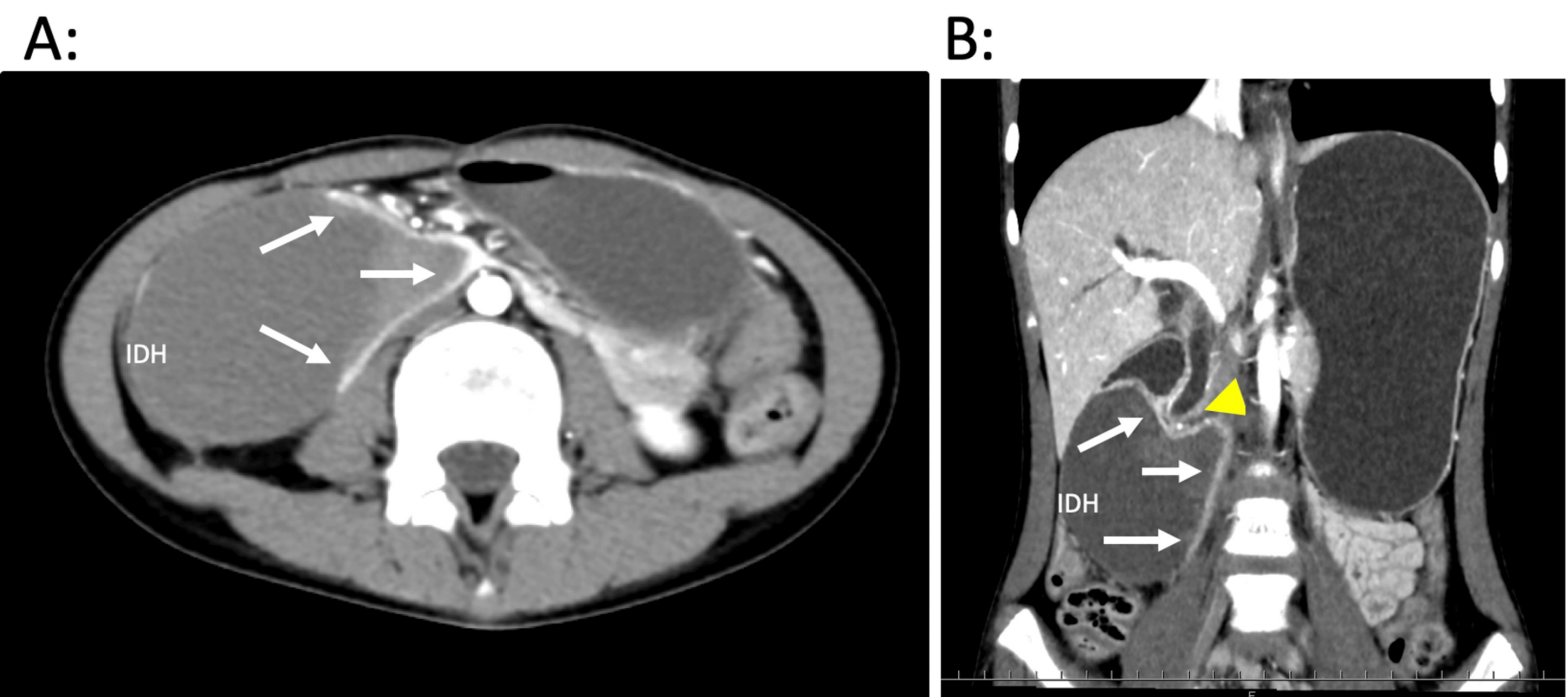
Contrast-enhanced abdominal computed tomography in (A) axial and (B) coronal views. The intramural duodenal hematoma, measuring 7.0 cm × 4.5 cm, compressed and obstructed the duodenum (arrow) and also compressed the bile duct (arrowhead).

**Figure 2:**
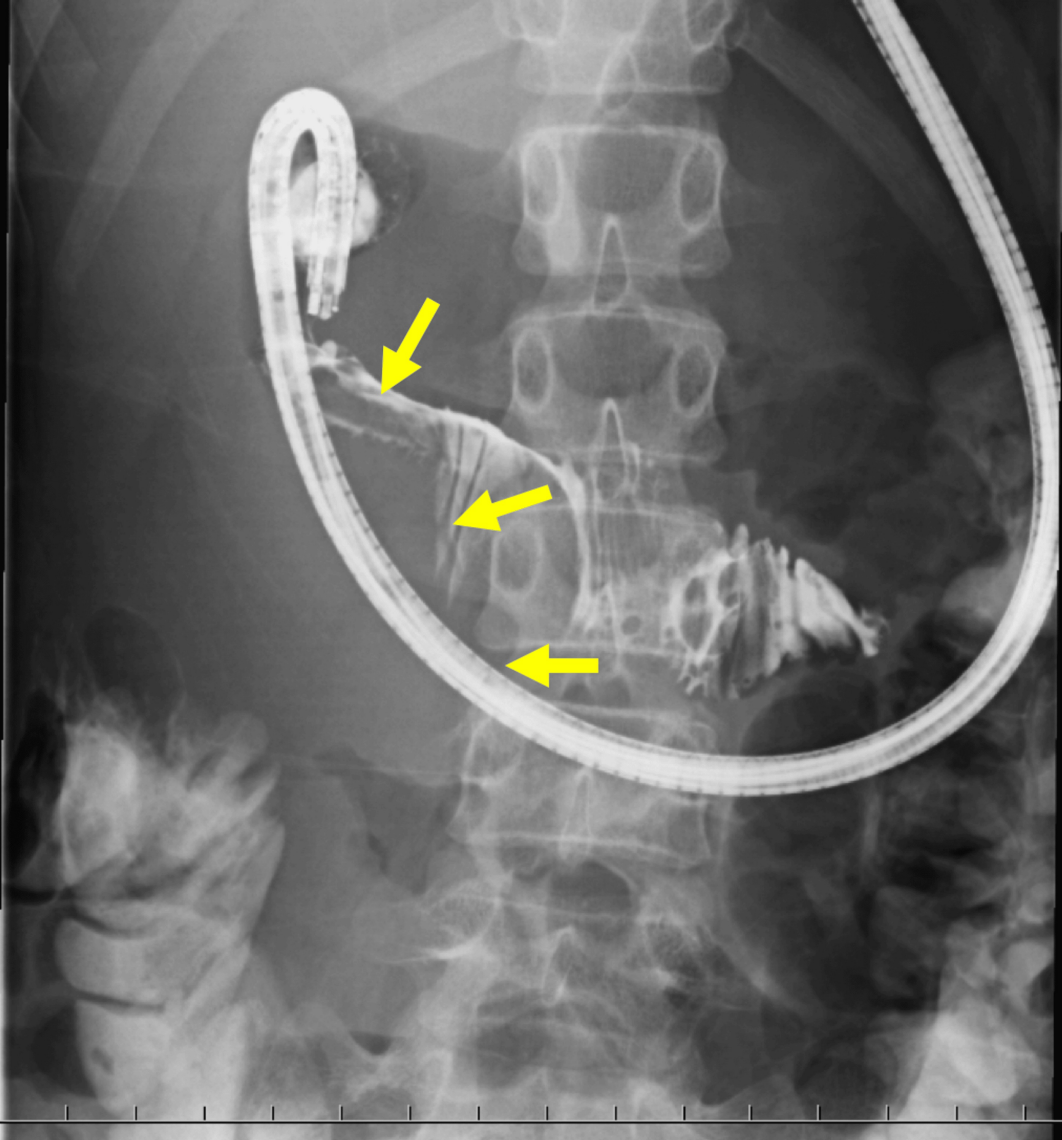
Esophagogastroduodenoscopy and contrast radiography. The intramural duodenal hematoma (arrow) compressed the descending portion of the duodenum.

**Figure 3:**
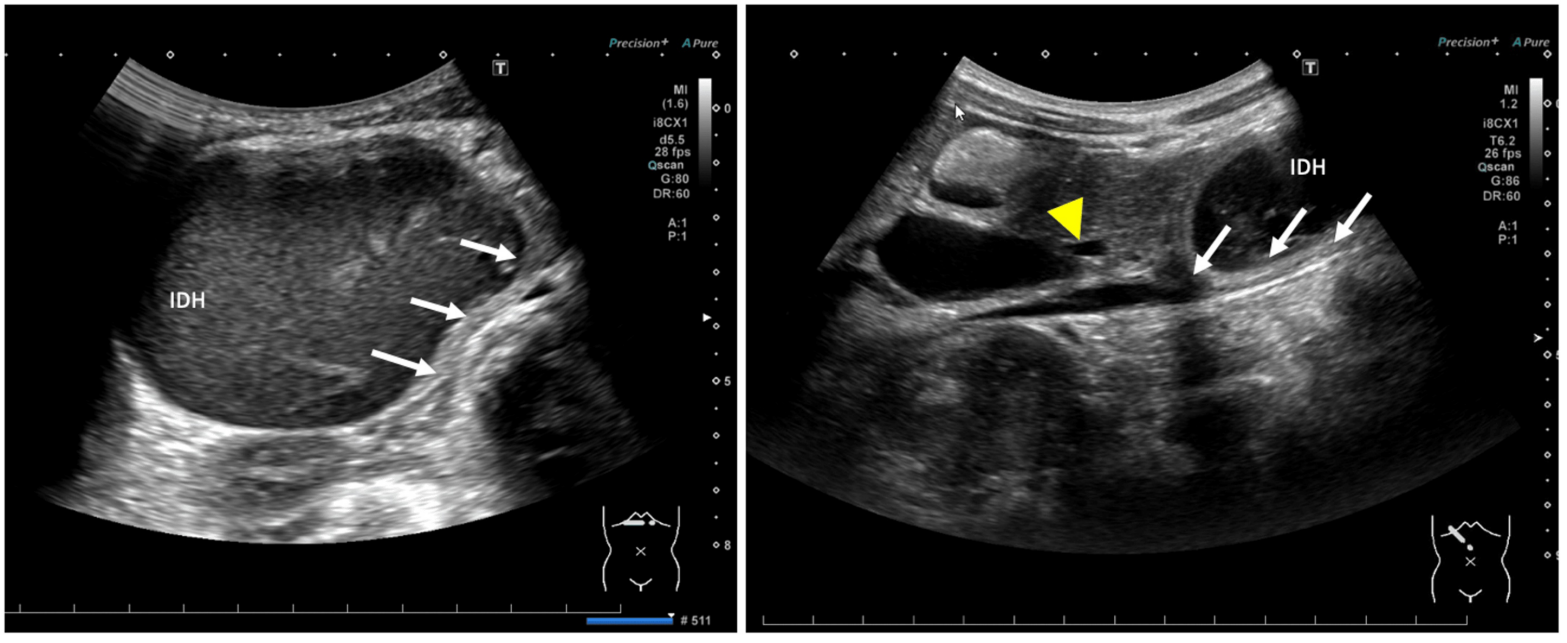
Abdominal ultrasonography. A hypoechoic mass (intramural duodenal hematoma) measuring 7.1 cm × 4.5 cm was compressing both the duodenum (arrow) and the common bile duct (arrowhead).

Contrast-enhanced abdominal computed tomography demonstrated an intramural hematoma (IDH) measuring 7.0 cm × 4.5 cm in the descending portion of the duodenum (Figure 1). No other organ damage was noted. An esophagogastroduodenoscopy and contrast radiography also detected obstruction of the duodenum (Figure 2). Conservative management was initiated with peripheral parenteral nutrition and nasogastric tube decompression. On day 11 post-admission, follow-up abdominal ultrasonography showed no significant reduction in the size of the hematoma (Figure 3). Therefore, surgical intervention was performed.

Operative procedure

**Figure 4:**
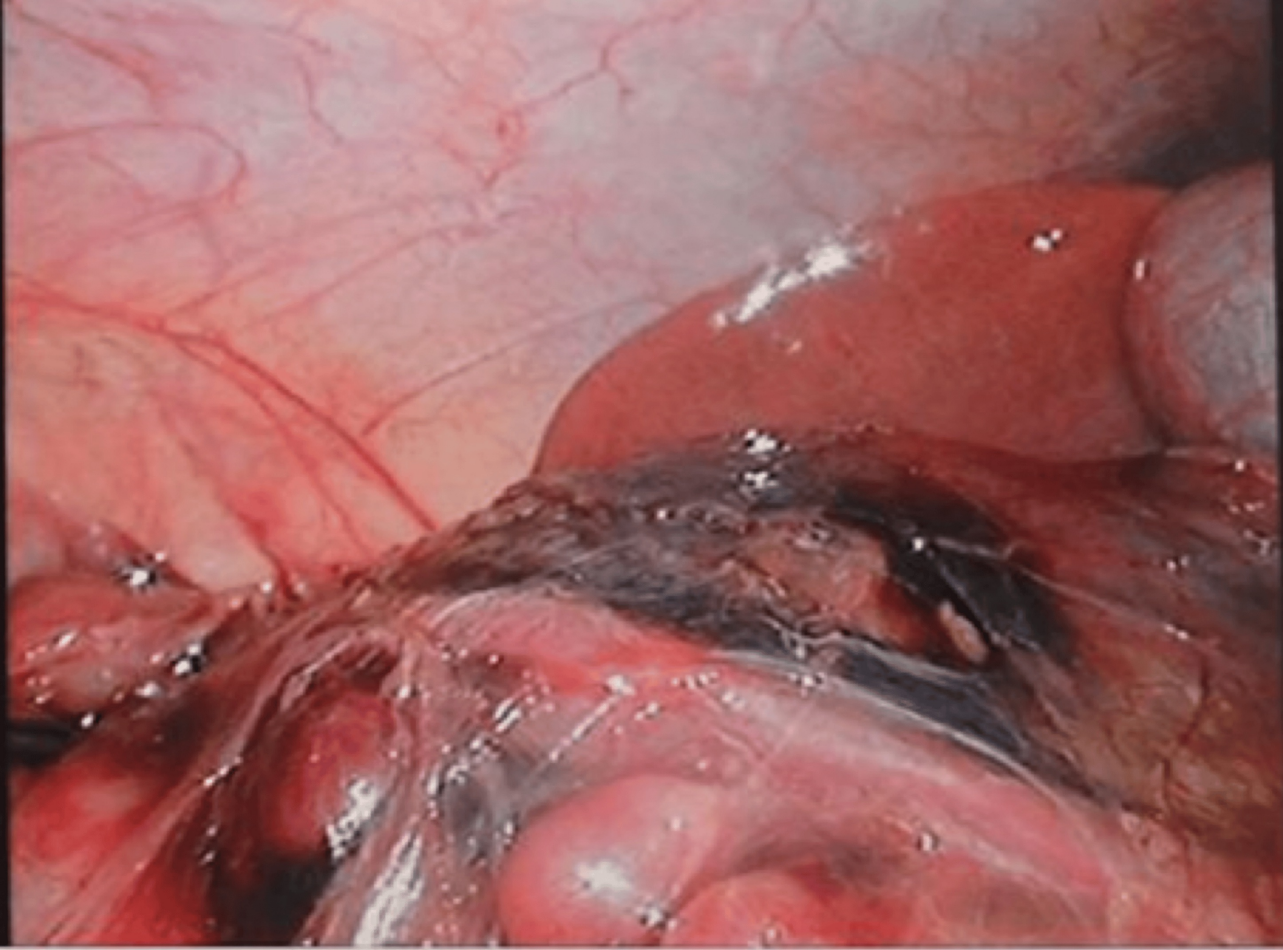
Operative findings. Note the exposed intramural duodenal hematoma wall after dissection of the mesentery of the transverse colon.

**Figure 5:**
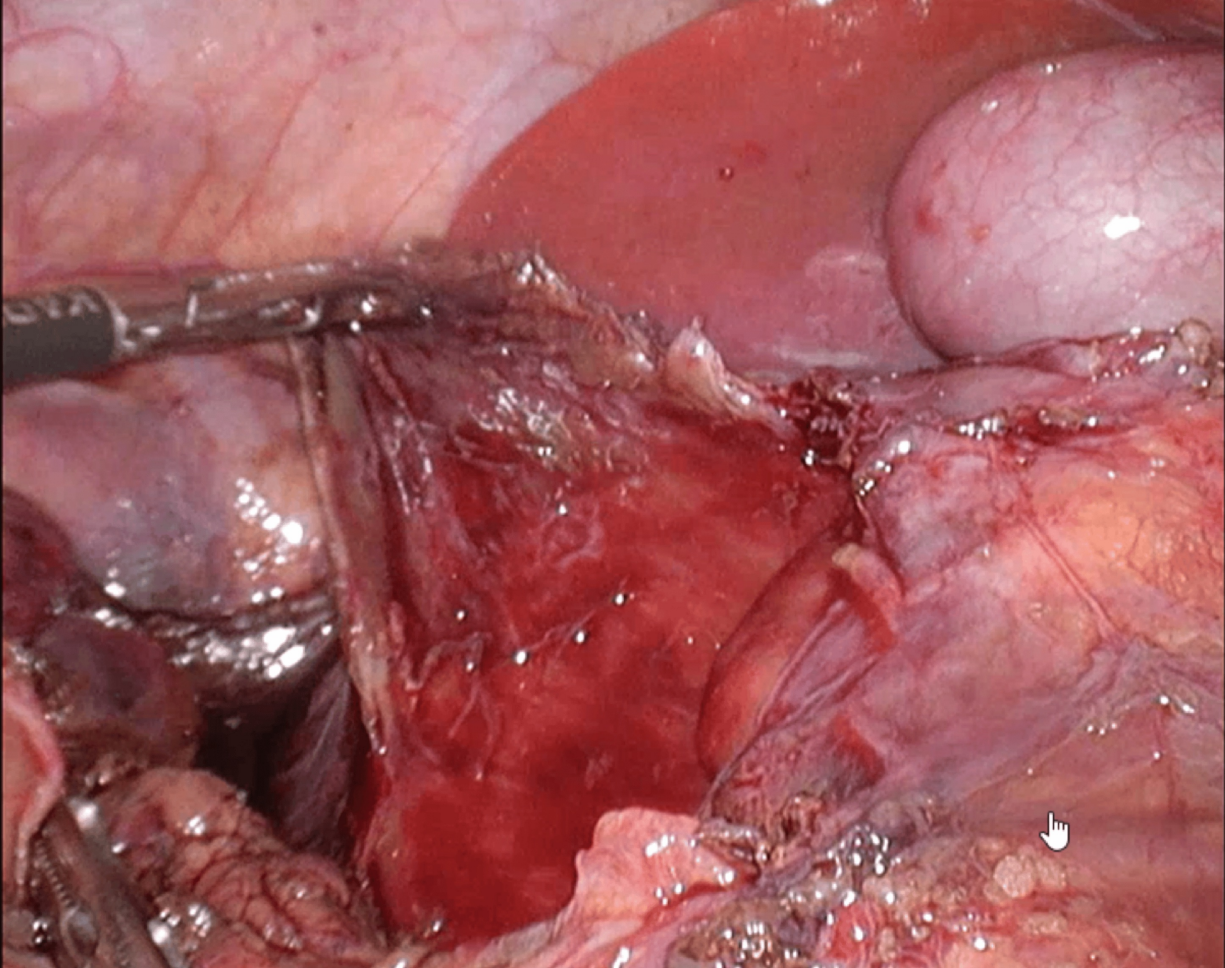
Operative findings. The intramural duodenal hematoma wall was incised longitudinally, the blood clot was removed, and the duodenal mucosa was identified.

**Figure 6:**
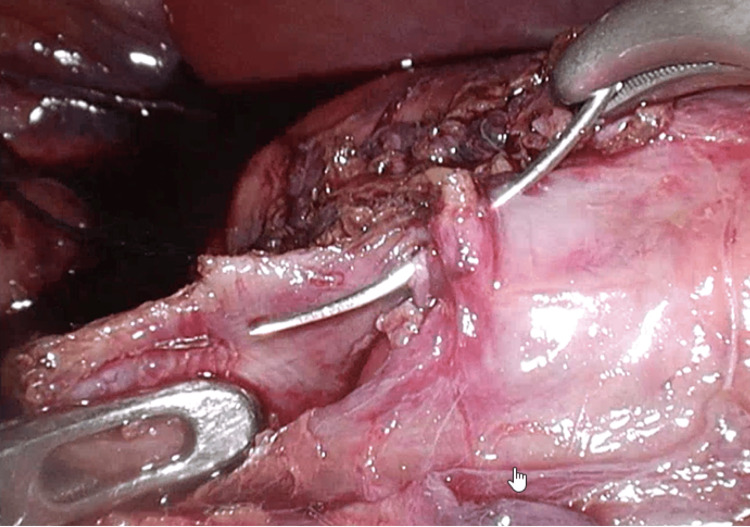
Operative findings. The hematoma wall (seromuscular layer of the duodenum) was sutured using 4-0 V-loc®. The suture was partially left open to allow for drainage.

Under general anesthesia, a 5-mm trocar was inserted through the umbilicus, and laparoscopy was performed to observe the abdominal cavity. A mass was identified in the descending portion of the duodenum (Figure 4). Three additional 5-mm trocars were placed for operation. The transverse mesocolon was opened to expose the duodenum with the IDH, and the serosa was widely opened to evacuate the blood clot (Figure 5). The duodenal mucosa was confirmed to be intact through the IDH cavity. The serosa layer of the duodenum was intracorporeally sutured and closed. Sutured closure was intentionally left incomplete to facilitate drainage (Figure 6). A 10 Fr J-VAC suction drain® was placed near the site.

The surgical drain was removed on postoperative day 6, and oral intake was initiated. The patient was discharged on postoperative day 13. The preoperatively elevated CK and BUN levels had returned to within normal ranges. The patient has been followed up for six months postoperatively, and no issues have arisen.

## Discussion

IDH is a rare disease in children. Its known causes include blunt abdominal trauma, bleeding disorders, anticoagulant therapy, malignancies, and iatrogenic events during esophagogastroduodenoscopy [1,2]. Approximately 5% of IDH cases in children are reported to be caused by blunt trauma, which can result not only from abdominal but also from chest trauma [3].

The proposed mechanism of IDH formation in cases of blunt abdominal trauma is that the duodenum, due to its anatomical position over the spine, and its fixation to the posterior abdominal wall and the ligament of Treiz, is subjected to external compression, which causes separation between the mucosal and submucosal layers [1,4].

Mahour et al. previously proposed the following criteria for performing conservative treatment: clinical, laboratory, and radiographic evidence that are absent of associated injuries to other organs, no excessive or continuous blood loss, and improvement of obstruction within 7 to 10 days [8]. The basic approach to conservative treatment consists of peripheral nutrition or total parenteral nutrition, and gastric decompression via a nasogastric tube [4-8].

As alternatives to avoid surgical intervention, ultrasound- or CT-guided drainage or endoscopic drainage by incising the duodenal mucosa near the hematoma have also been reported [9-11].

When conservative management proves ineffective, surgical intervention should be considered. However, since surgery can be highly invasive, especially in pediatric patients, laparoscopic surgery is considered preferable [1]. Nevertheless, in cases with associated organ injuries, particularly pancreatic trauma, open surgery may be necessary as laparoscopic surgery may be insufficient.

In blunt trauma-induced pediatric IDH, conservative management should be the first-line treatment while evaluating for potential associated organ injuries in parallel. In surgical management, laparoscopic exploration should be performed initially to confirm that no other organ has been injured. If laparoscopic surgery is deemed feasible, additional small-diameter operative trocars (3-5 mm in diameter) can be placed to proceed with the intervention. In addition, the serosal suturing performed after evacuation of the hematoma within the IDH lumen was intentionally left partially open. This method was considered effective in preventing postoperative reaccumulation of the hematoma.

## Conclusions

Pediatric IDH following blunt trauma can often be managed conservatively. However, surgical intervention may be necessary if obstruction persists. Laparoscopic evacuation with partial closure and drainage is effective and minimally invasive, allowing safe recovery and minimizing complications in appropriately selected cases.
